# Impact of COVID-19 Infection on Quality of Sleep

**DOI:** 10.7759/cureus.18182

**Published:** 2021-09-22

**Authors:** Ayesha A Choudhry, FNU Shahzeen, Sara A Choudhry, Narjis Batool, Fatir Murtaza, Anum Dilip, Murk Rani, Aakash Chandnani

**Affiliations:** 1 Medicine, Fatima Jinnah Medical University, Lahore, PAK; 2 Internal Medicine, Jinnah Sindh Medical University, Karachi, PAK; 3 Internal Medicine, Quaid-e-Azam Medical College, Bahawalpur, PAK; 4 Internal Medicine, Gomel State Medical University, Gomel, BLR; 5 Internal Medicine, Chandka Medical College, Karachi, PAK

**Keywords:** corona virus disease, sleep pattern, insomnia, sars-cov-2 (severe acute respiratory syndrome coronavirus -2), covid-19

## Abstract

Introduction: Some studies have highlighted the effect of COVID-19 infection on the quality of sleep; however, the data is limited. In this study, we investigated the prevalence of insomnia in patients who recently recovered from the COVID-19 infection to evaluate the prevalence and extent of its impact.

Methods: This longitudinal study was conducted from January 2021 to March 2021. A total of 500 patients admitted to the intensive care unit or isolation unit of COVID-19 were included in the study at the time of their discharge. The pre-COVID-19 sleep quality of the participants was inquired using the Pittsburgh Sleep Quality Index (PSQI). Post-COVID sleep quality was assessed at a 30-day follow-up. Sleep quality was considered poor if the global score was ≥5. Participants that failed to follow up were not included in the study.

Results: The mean PSQI score was significantly higher in the post-COVID-19 group compared to the pre-COVID-19 group (6.28 ± 2.11 vs. 3.22 ± 0.80; p-value <0.0001). The percentage of participants with a PSQI score of ≥5 was significantly higher in the post-COVID-19 group compared to the pre-COVID-19 group (45.1% vs. 12.1%; p-value <0.0001).

Conclusion: Insomnia has a significant prevalence in recovered COVID-19 patients after 30 days of follow-up. Hence, patients need to be counseled to follow up in case they experience poor sleep. To avoid the long-term negative impact on patients experiencing insomnia, timely identification and treatment are important.

## Introduction

Coronavirus disease 2019 (COVID-19) is a respiratory illness that resulted in an outbreak in December 2019 and progressed to pose a global pandemic. This virus is one of the six species under a family of enveloped, single-stranded RNA viruses known as the Coronaviridae [1]. Severe acute respiratory syndrome coronavirus 2 (SARS-CoV-2) has emerged with different variants, however, four are of major concern; alpha, beta, gamma, and the delta variant [2]. These variants have differing transmission, virulence, and mortality rates.

The incubation period ranges from five to six days post-exposure but can take up to 14 days. Although the individuals are asymptomatic during this phase, they are still contagious and can transmit the virus to others [3]. Its presentation can range from asymptomatic infection to fatal respiratory illness. Mild to moderate involvement is observed in 80% of the infected individuals. Risk factors for severe disease include infants, individuals >65years, and those with preexisting comorbidities [4]. Commonly reported symptoms are fever, shortness of breath, myalgias, headaches, cough, runny nose, and fatigue [5]. On average, the majority of the mild to moderate categories of COVID-19 infections last for 11.5 ± 5.7 days in duration [6]. Another symptom that is rarely studied is insomnia post-COVID-19. Jaharmi et al. stated that sleep disturbance is very common in patients who had contracted COVID-19 [7]. Insomnia and decreased quality of sleep in COVID-19 patients may be due to physical pain and side effects of medications administered for the treatment of the virus [8].

In this study, we shall investigate the prevalence of insomnia as a post-COVID-19 symptom in patients who have recently recovered from the infection. Furthermore, we aim to study the extent of its impact on this population.

## Materials and methods

This longitudinal study was conducted from January 2021 to March 2021. Five hundred patients, who were being discharged from the Intensive care unit (ICU) after recovery from COVID-19, were enrolled in this study. Participants of either gender, between the age group of 18 to 60 years, were enrolled via consecutive convenient non-probability sampling. Patients who were taking medication for anxiety, depression, or anxiety before contracting COVID-19 were excluded from the study. The participants were informed beforehand that their participation was voluntary and they could back off from the study at any point. Ethical review approval was taken from Jinnah Sindh Medical University (JSMU/IRB/2021/07) before the enrollment of patients.

The pre-COVID-19 sleep quality of the participants was inquired. The quality of sleep was assessed using the Pittsburgh Sleep Quality Index (PSQI), which measures the quality and pattern of sleep. The score ranges from 0-21. Sleep quality is considered poor if the global score is ≥5. Its internal consistency and reliability coefficient (Cronbach's alpha) is 0.83 [9]. Participants were asked to return to follow up after 30 days and PSQI was repeated. Fifty-five participants were not available for follow-up. Only those participants that completed the follow-up were included in the final analysis.

Data analysis was done using the Statistical Package for the Social Sciences (SPSS), version 22.0 (IBM Corp., Armonk, NY, USA). Patients’ demographics were calculated as frequencies and percentages. Numerical data was presented as mean and standard deviation. A comparison of the PSQI score was made for pre-COVID-19 and post-COVID-19 data using chi-square or t-test, as appropriate. A p-value of less than 0.05 was considered statistically significant when comparing the two groups.

## Results

The median days of hospitalization were four days. In total, 445 of the 500 discharged post-COVID-19 patients were enrolled in the study after excluding the 55 patients that were lost to follow-up. The mean age of participants was 42 ± 10 years. Out of the total participants, there were 291 (65.3%) male participants. Patients were followed up 30 days after their recovery from COVID-19 (Table 1).

**Table 1:** Characteristics of participants at the time of discharge CRP: C-reactive protein, DM: diabetes mellitus, ESR: erythrocyte sedimentation rate, IU: international unit, LDH: lactate dehydrogenase, mg/L: milligram per liter, mm/hr: millimeters per hour

Characteristics at the time of discharge	Value (n=445)
Age (in years)	42 ± 10
Male (%)	291 (65.3%)
Smoking (%)	101 (22.6%)
Hypertension (%)	112 (25.1%)
DM type 2 (%)	118 (26.5%)
Median number of days at hospital	4
CRP (mg/L)	12.1 ± 3.7
LDH (IU)	282.3 ± 89.2
ESR (mm/hr)	13.1 ± 3.1

The mean PSQI score was significantly higher in the post-COVID-19 group compared to the pre-COVID-19 group (6.28 ± 2.11 vs. 3.22 ± 0.80; p-value <0.0001). The percentage of participants with a PSQI score of ≥5 was significantly higher in the post-COVID-19 group compared to the pre-COVID-19 group (45.1% vs. 12.1%; p-value <0.0001) (Table 2).

**Table 2:** Comparison of PSQI score before and 30 days after COVID-19 PSQI: Pittsburgh Sleep Quality Index

PSQI score	Pre-COVID-19 (n=445)	30 days Post-COVID-19 (n=445)	p-value
Mean score	3.22 ± 0.80	6.28 ± 2.11	<0.0001
Participants with ≥5 score	54 (12.1%)	201 (45.1%)	<0.0001

## Discussion

In our study, the mean PSQI score was significantly higher in the post-COVID-19 group compared to the pre-COVID-19 group. According to our study, 45.1% of the participants who contracted COVID-19 reported a PSQI score of >5, signifying the poor quality of sleep at a 30-day post-recovery follow-up, whereas only 12.1% of the participants had reported poor quality of sleep before contracting the infection. This accounts for a 33% increase in the participants experiencing insomnia after COVID-19.

There are several studies that have reported insomnia as one of the symptoms experienced with long COVID. A six-month follow-up study from China on 1733 patients stated that 26% of the participants reported sleep difficulties after recovering from COVID-19 [10]. A study was conducted on 507 individuals to compare sleep disturbances using Insomnia Severity Index (ISI) between non-COVID, COVID positive, and post COVID patients. The study demonstrated that patients with long COVID showed a significant prevalence of insomnia along with an overall reduction in the quality of life (QOL) [11]. In a case series conducted on post-COVID-19 patients discharged from the University of Virginia ICU, 22% of patients reported new-onset insomnia at a six-week follow-up when assessed using ISI [9].

Various factors can contribute to the decrease in the quality of sleep in these patients. In a four-week follow-up on COVID-19 survivors, Mazza et al. found that 28% were suffering from post-traumatic stress disorder, 31% from depression, 42% from anxiety, and 40% from insomnia [12]. The aforementioned mental health conditions are known causes of sleep disturbances and their co-existence in long COVID plays a role in the development of insomnia [13-15]. Furthermore, illness leads to the aggravation of already high baseline stress due to the ongoing social distancing restrictions, travel restrictions, economic-related stress, COVID-related worries, and loneliness [16]. 

Insomnia is a syndrome that negatively impacts an individual’s life. It can lead to impaired cognitive functioning, lower productivity, an increase in irritability, and poor interpersonal relations [17]. Moreover, studies have shown that chronic insomnia increases the susceptibility for pain disorders and gastrointestinal distress. If left untreated, it also puts the patient at risk for hypertension and heart diseases [17]. Besedovsky et al. mentioned that prolonged sleep deprivation can lead to the production of pro-inflammatory cytokines which induces a state of mild inflammation and decreases immunity [18]. All these manifestations lead to a decrease in QOL and delayed recovery; hence, warranting the need to appropriately manage it [19].

Based on our findings, we suggest that individuals who have recovered from COVID-19 should be screened for long COVID symptoms along with neuropsychological screening on follow-ups. Especially those who report sleep disturbances should be evaluated for coexisting depression and/or anxiety. These patients should be adequately treated for insomnia and any underlying causes. There are multiple options available including melatonin, benzodiazepines, and zolpidem which can be tailored according to the patient’s specifics. Patients suffering from underlying psychiatric disorders may also benefit from cognitive-behavioral therapy.

Limitations 

First, the study was conducted in a single tertiary care hospital, hence care should be taken while inferring the result to the general population. Our study does not include patients suffering from mild COVID-19 as all our data has been collected from patients that were previously receiving inpatient care. More inclusive data may be required to critically comment on the true prevalence of insomnia in long COVID. Also, there was no record of participants' pre-COVID-19 sleep pattern, hence there might be a possibility of a recall bias. 

## Conclusions

In our study, patients who have suffered from COVID-19 have a higher prevalence of insomnia compared to before the illness. To avoid the long-term negative impact on patients experiencing insomnia, timely identification and treatment is important. Co-existing neuropsychological disorders should also be screened for and managed accordingly. This intervention can impact both the psychological and physical recovery of the patient.
